# Sodium Bicarbonate and Time-to-Exhaustion Cycling Performance: A Retrospective Analysis Exploring the Mediating Role of Expectation

**DOI:** 10.1186/s40798-023-00612-5

**Published:** 2023-07-31

**Authors:** William H. Gurton, Guilherme G. Matta, Lewis Anthony Gough, Mayur Krachna Ranchordas, David G. King, Philip Hurst

**Affiliations:** 1 School of Psychology and Life Sciences, Canterbury Christ Church University, Canterbury, UK; 2 Sport and Physical Activity Research Centre, College of Health, Wellbeing and Life Sciences, Sheffield Hallam University, Sheffield, UK; 3 Human Performance and Health Research Group, Centre for Life and Sport Sciences, Birmingham City University, Birmingham, UK; 4 School of Biosciences and Medicine, University of Surrey, Guildford, UK

**Keywords:** Ergogenic aids, Placebo effect, Beliefs, Extracellular buffering, High-intensity exercise

## Abstract

**Background:**

Research has shown that ingesting 0.3 g·kg^−1^ body mass sodium bicarbonate (NaHCO_3_) can improve time-to-exhaustion (TTE) cycling performance, but the influence of psychophysiological mechanisms on ergogenic effects is not yet understood.

**Objective:**

This study retrospectively examined whether changes in TTE cycling performance are mediated by positive expectations of receiving NaHCO_3_ and/or the decline in blood bicarbonate during exercise.

**Methods:**

In a randomised, crossover, counterbalanced, double-blind, placebo-controlled design, 12 recreationally trained cyclists (maximal oxygen consumption, 54.4 ± 5.7 mL·kg·min^−1^) performed four TTE cycling tests 90 min after consuming: (1) 0.3 g·kg^−1^ body mass NaHCO_3_ in 5 mL·kg^−1^ body mass solution, (2) 0.03 g·kg^−1^ body mass sodium chloride in solution (placebo), (3) 0.3 g·kg^−1^ body mass NaHCO_3_ in capsules and (4) cornflour in capsules (placebo). Prior to exercise, participants rated on 1–5 Likert type scales how much they expected the treatment they believe had been given would improve performance. Capillary blood samples were measured for acid-base balance at baseline, pre-exercise and post-exercise.

**Results:**

Administering NaHCO_3_ in solution and capsules improved TTE compared with their respective placebos (solution: 27.0 ± 21.9 s, *p* = 0.001; capsules: 23.0 ± 28.1 s, *p* = 0.016). Compared to capsules, NaHCO_3_ administered via solution resulted in a higher expectancy about the benefits on TTE cycling performance (Median: 3.5 vs. 2.5, *Z* = 2.135, *p* = 0.033). Decline in blood bicarbonate during exercise was higher for NaHCO_3_ given in solution compared to capsules (2.7 ± 2.1 mmol·L^−1^, *p* = 0.001). Mediation analyses showed that improvements in TTE cycling were indirectly related to expectancy and decline in blood bicarbonate when NaHCO_3_ was administered in solution but not capsules.

**Conclusions:**

Participants’ higher expectations when NaHCO_3_ is administered in solution could result in them exerting themselves harder during TTE cycling, which subsequently leads to a greater decline in blood bicarbonate and larger improvements in performance.

**Key Points:**

- Ingesting 0.3 g·kg^−1^ body mass sodium bicarbonate in solution and capsules improved time-to-exhaustion cycling performance
- Positive expectancy about the benefits of sodium bicarbonate and decline in blood bicarbonate were higher when sodium bicarbonate was administered in solution compared with capsules
- Improvements in time-to-exhaustion cycling performance for sodium bicarbonate administered in solution were related to expectancy and the enhanced extracellular buffering response

## Introduction

Sodium bicarbonate (NaHCO_3_) is suggested to be an effective ergogenic aid during high-intensity, short duration exercise [1, 2]. Ingesting 0.3 g·kg^−1^ body mass (BM) NaHCO_3_ 60–90 min prior to exercise increases blood bicarbonate concentration by ~5 to 6 mmol·L^−1^ and in turn, elevates pH gradient between intracellular and extracellular compartments [3]. This allows for greater efflux of hydrogen cations (H^+^) from active musculature into circulation, reducing muscle pH during high-intensity exercise [4]. Although the deleterious effect of intramuscular acidosis on muscle fatigue is disputed [5, 6], declining muscle pH during intense exercise is believed to inhibit metabolic processes required for energy production from anaerobic pathways [7–9]. Enhanced extracellular buffering after NaHCO_3_ ingestion could therefore sustain anaerobic energy production and improve high-intensity exercise performance.

Whilst there is empirical support for the potential physiological mechanisms underlining the benefits of NaHCO_3_ on sports performance, it is clear that findings are equivocal [2]. Researchers have shown NaHCO_3_ administered in solution to improve time-to-exhaustion (TTE) cycling performance [10–12], whereas others have reported no improvements when NaHCO_3_ was given in capsules [13–15]. One reason for these inconsistencies between ingestion strategies could relate to participants’ expectancy, which is an anticipation of a future event that is thought to induce the placebo effect [16]. In short, placebo effects are positive outcomes arising from an individuals’ expected and/or learned responses to a treatment [17]. A plethora of research has identified that expectations about dietary supplements contributes to improvements in sporting performance [18]. That is, when an athlete ingests a supplement and expects it to be performance enhancing they are more likely to report improvements in performance than when they do not expect it to improve performance [18]. McClung & Collins [19] reported that when participants received NaHCO_3_ and expected to receive NaHCO_3_, their 1000-m running time trial improved more than when participants received NaHCO_3_ but expected a placebo. Furthermore, they found similar improvements in performance for participants receiving a placebo or NaHCO_3_ when they expected NaHCO_3_. Similar results have been reported elsewhere for NaHCO_3_ [20] and other dietary supplements such as caffeine and carbohydrates [21, 22]. These findings demonstrate that athletes’ expectations about ergogenic aids can influence whether positive outcomes are observed.

Since the placebo effect is a complex phenomenon that can be described from neurobiological and psychosocial perspectives [23], it is reasonable to suggest that any expectations about NaHCO_3_ may interact with physiological mechanisms underpinning pharmacological properties [18]. One example could be the potential interaction of expectation and decline in blood bicarbonate during exercise on performance. Irrespective of pre-exercise changes in blood bicarbonate after NaHCO_3_, it is possible that decline in blood bicarbonate during exercise directly infers the degree of extracellular buffering (i.e., amount of H^+^ removed from muscles) and thus the capacity for NaHCO_3_ to improve exercise performance [24]. Any relationship between expectancy and decline in blood bicarbonate might be explained by response expectancy theory [16]. In brief, this posits that predictions about responses can induce placebo effects, and in turn affect physiological processes [16, 25]. Theoretically, athletes that expect they have received NaHCO_3_, and that it will improve performance, might exert themselves to a greater extent during exercise due to psychologically manipulated expectations (i.e., reduced feelings of fatigue) [26] and thus, augment the extracellular buffering capacity of NaHCO_3_ (thus leading to greater decline in blood bicarbonate) [27]. However, no study has examined the interaction between expectations and decline in blood bicarbonate following NaHCO_3_ ingestion, therefore further work is warranted to determine its impact on sports performance.

Recently, we reported that participants are less likely to detect NaHCO_3_ administered in capsules than solution (67% vs. 17% ‘unsure’ ratings) partially due to the ‘poor’ taste of solution beverages [28]. Importantly, these differences in blinding between NaHCO_3_ ingestion strategies may influence participants’ expectations of positive outcomes (i.e., greater response expectancy after identifying NaHCO_3_) that mean researchers administering NaHCO_3_ in solution during randomised controlled trials overestimate its ‘true’ pharmacological effectiveness for improving exercise performance. Therefore, we retrospectively examined data from a previous study [28] to (1) determine the influence of expectation and decline in blood bicarbonate following NaHCO_3_ administered in solution and capsules on TTE cycling performance, and (2) examine if changes in performance are mediated by the expectation of receiving NaHCO_3_ and decline in blood bicarbonate.

## Methods

### Study Design

A randomised, crossover, double-blind, placebo-controlled, counterbalanced design was employed. Participants attended five laboratory visits to perform TTE cycling tests (1 x familiarisation, 4 x experimental trials). To control for order effects, participants were randomly assigned to receive one of four treatments (NaHCO_3_ and placebo administered in solution and capsules) at each visit in a balanced fashion using a Latin square sequence by a member of the research team not involved with data collection.

### Participants^1^

Our sample size calculation conducted on G*Power (version 3.1.9.4) revealed that 12 participants were needed to achieve statistical power (*β* = 0.80; *α =* 0.05). This was based on using repeated measures analysis of variances (within-factors) to analyse differences in TTE cycling performance, with an expected medium effect size (partial eta squared, *η*_*p*_^*2*^ = 0.06). Correlation between repeated measures was estimated from previous reliability data for a similar TTE cycling test [29]. To account for typical dropout rates, sixteen recreationally trained cyclists were recruited to participate in the study; however, two did not meet inclusion criteria, one withdrew because of injury and one withdrew due to side-effects after NaHCO_3_ ingestion. Therefore, 12 recreationally trained cyclists (9 men, 3 women; height, 176.3 ± 5.6 cm; body mass, 69.4 ± 8.1 kg; age, 29.3 ± 6.7 years; maximal oxygen consumption, 54.4 ± 5.7 mL·kg^−1^·min^−1^) completed study procedures. Inclusion criteria stipulated that participants were: i) aged between 18 and 40 years, ii) performing at least 3 h cycling per week, iii) unaware of the benefits of NaHCO_3_ on sport performance, iv) not using extra- or intracellular buffering aids during training (i.e., NaHCO_3_, beta-alanine), v) not intolerant to cornflour, and vi) not diagnosed with a medical condition that could impact high-intensity exercise. To control for any small confounding effect of menstrual cycle on anaerobic exercise performance [30], we asked female participants to record their menstrual cycle (using a calendar-based method) to ensure experimental trials occurred during the same phase (follicular: 1–14 d, or luteal: 14 d to start of next cycle). Ethical approval was gained from the lead authors’ Institutional Ethics Committee (ETH2021-0198). Research procedures were conducted in accordance with the revised Declaration of Helsinki (2013). All participants provided written informed consent prior to commencing study procedures.

### Supplementation Protocol

Across four experimental trials, participants ingested: (1) 0.3 g·kg^−1^ BM NaHCO_3_ in 5 mL·kg^−1^ BM solution (SB-SOL), (2) 0.03 g·kg^−1^ BM sodium chloride in 5 mL·kg^−1^ BM solution (placebo; PL-SOL), (3) 0.3 g·kg^−1^ BM NaHCO_3_ within size 0 vegetarian capsules (SB-CAP), or (4) an equal number of capsules that contained cornflour (placebo; PL-CAP). Pilot testing revealed that 0.03 g·kg^−1^ BM sodium chloride provided the best taste-match with 0.3 g·kg^−1^ BM NaHCO_3_ in solution, whereas other commonly used doses (i.e., 0.07 g·kg^−1^ BM) were deemed too ‘salty’ [28]. Cornflour was chosen as the placebo for capsule trials as it is an inert substance that effectively blinds NaHCO_3_ supplementation [28]. Solution treatments were prepared in 4 mL·kg^−1^ BM double-strength sugar free orange squash (Sainsbury’s, UK) and 1 mL·kg^−1^ BM water, before being chilled (~12°C) for 1 h prior to consumption [28]. All capsules (Your Supplements, Stockport, UK) were manually filled using a capsule filling device (ALL-IN Capsule, USA) and each capsule contained approximately either 0.9 g NaHCO_3_ (Health Leads Ltd, UK) or 0.4 g cornflour (Sainsbury’s, UK). Capsule treatments were given to the nearest number of whole capsules (mean ± SD = 24 ± 3; range = 20–28) in an equal volume of squash/water consumed during sodium bicarbonate solution (SB-SOL) and placebo solution (PL-SOL) trials. Treatments were prepared by a member of the research team not involved with data collection and solution treatments were administered in opaque bottles to prevent participants visually distinguishing between them. Participants were given a 10-min period to consume treatments, alongside ingesting a carbohydrate-rich meal (1.5 g·kg^−1^ BM carbohydrates; toasted bread/jam, cereal bars) over a 30-min period to minimise the risk of gastrointestinal (GI) discomfort [31].

### Blood Sampling

Finger prick capillary blood samples were taken to measure acid-base balance and lactate. To measure acid-base balance, 95 μL samples were collected into blood gas capillary tubes (Vitrex, Herlev, Denmark) and transferred into single-use i-STAT G3+ cartridges (Abbott, Illinois, USA). Samples were analysed for pH, bicarbonate and base excess using a portable blood gas analyser (i-STAT 1, Abbott, Illinois, USA), which has previously demonstrated excellent accuracy and precision for blood pH and bicarbonate (intraclass correlation coefficients, ICC = 0.88, 0.86) [32]. Additional 10 μL blood samples were collected into haemolysing cups (EKF Diagnostics, Cardiff, UK) and analysed for lactate using a Biosen C-Line (EKF Diagnostics; coefficient of variation < 1.5%, at a value of 12 mmol·L^−1^).

### Questionnaires

Participants completed treatment assignment questionnaires that required them to indicate which treatment they thought had been administered (see Gurton et al. [28] for data) and rate on 1–5 Likert-type scales how much they expected this treatment would improve their performance (“1” = no expectations at all, “5” = extremely high expectations). This was adapted from a 44-item Likert-type scale previously used to assess expectancies in psychological research that has been suggested to show good reliability (coefficient alphas, 0.81–0.89) [33]. Participants also completed 100-mm visual analogue scales (0” = no symptom, “100” = severest symptom) to quantify aggregate Gastrointestinal (GI) discomfort for eight common side-effects [12].

### Procedures

Participants attended the laboratory in a 3 h post-prandial state having avoided strenuous exercise and alcohol for 24 h. Caffeine consumption was also prohibited 12 h prior to testing. Nutritional intake was recorded via self-report food diaries for 24 h before the first trial and replicated prior to subsequent sessions (*n* = 11, one participant lost diary). Experimental trials were separated by 3–7 d to allow for appropriate washout of nutritional treatments. Testing was conducted at a similar time of day (± 2 h) to control for the confounding effects of circadian rhythms on exercise performance [34].

During the initial laboratory visit, anthropometric measures were recorded before participants performed a graded cycling test on an electronically braked SRM ergometer (Schoberer Rad Meßtechnik, Germany). Gaseous exchange was collected using a breath-by-breath metabolic analyser (Vyntus CPX, CareFusion GmbH, Germany). Participants completed a 5 min warm-up at 70 W and a self-selected cadence (60–90 rev·min^−1^). Power output during the first stage was prescribed according to participants’ fitness level, with increments of +5 W applied every 15 s such that volitional exhaustion occurred within ~8 to 12 min [35]. Average power output across the final 2 min (W_peak_) was used to calculate workload for TTE cycling tests [29]. Maximal oxygen consumption was recorded as the highest 30 s average for oxygen uptake [35]. Following 30 min recovery, participants were familiarised to the TTE cycling test. Workload was adapted from the 110% W_peak_ chosen by Saunders et al. [29] to cause fatigue within ~5 min, as this duration has been shown to elicit the greatest ergogenic benefits for NaHCO_3_ ingestion [11]. Participants selected preferred bike dimensions (replicated during subsequent TTE tests) and completed a 5 min warm-up at 1.5 W·kg^−1^ BM. This intensity was chosen to prepare participants for TTE cycling, without diminishing extracellular buffering capacity [36]. Power output was then increased across 60 s (increments every 15 s) until desired workload was achieved, at which point TTE cycling commenced. Participants' cadence was visible, but time was concealed. Exercise was terminated when participants failed to maintain cadence > 60 rev·min^−1^ for 5 s despite verbal encouragement.

On arrival to the laboratory during experimental trials, baseline capillary blood samples were taken and GI discomfort questionnaires were completed. Participants then consumed nutritional treatments (SB-SOL, SB-CAP, PL-SOL or PL-CAP) and the carbohydrate-rich meal. Additional GI discomfort questionnaires were filled in at 30-min and 60-min post-consumption. Capillary blood samples were performed prior to exercise, which was 85 min post-consumption. At this point, participants repeated GI discomfort questionnaires and completed treatment assignment questionnaires. TTE cycling tests commenced 90 min after consuming supplements and were performed as described during the familiarisation visit. Final capillary blood samples and GI discomfort questionnaires were repeated post-exercise.

### Statistical Analysis

Data were analysed using SPSS version 26.0 (IBM, New York, USA). Descriptive data are presented as mean ± SD (unless otherwise stated) and the *α*-level of statistical significance was set at *p* < 0.05. Normality was determined from Shapiro-Wilk tests and sphericity was assessed using Mauchly’s test, with violations corrected via Greenhouse-Geisser. Two-way repeated measures analysis of variance (ANOVA) were used to establish significant treatment * time interactions for blood analyses and least significant difference post-hoc pairwise comparisons were conducted to determine treatment differences at each time point [37]. One-way repeated measures ANOVA were used to determine whether there were any differences between treatments for TTE cycling performance and decline in blood bicarbonate. Effect sizes for ANOVA main effects and interactions are reported as partial eta squared (*η*_*p*_^*2*^), with values of 0.1, 0.25 and 0.4 representing small, medium and large effects, respectively [38]. Between treatment effects sizes (*g*) were calculated by dividing mean difference by pooled SD and applying Hedges *g* bias correction to account for the small sample size [39]. These were interpreted as trivial (≤ 0.20), small (0.20–0.49), moderate (0.50–0.79) or large (≥ 0.80) [38]. Friedman tests were conducted on non-normally distributed data (i.e., expectancy, aggregate GI discomfort) with median and *Z* values reported for between treatment comparisons. Non-normally distributed effect sizes (*r*) were calculated from *Z*/√n, with 0.10, 0.24 and 0.37 considered small, medium, and large, respectively [40].

To examine whether changes in TTE were mediated by expectancy of NaHCO_3_ and decline in blood bicarbonate, we used Process v4.0 [41] SPSS macro (model 6), which simultaneously tests direct and indirect effects in a serial mediation model. Briefly, serial mediation was chosen as we hypothesised that expectations would affect decline in blood bicarbonate that in turn, would influence sport performance; as opposed to parallel mediation approaches that would not consider the influence of expectations on decline in blood bicarbonate [42]. Direct effects are the effects of the predictor variable (i.e., treatment) on an outcome variable (i.e., TTE) that occur separately to each mediator, while indirect effects are the effects of treatment on TTE via expectancy and decline in blood bicarbonate. Given that the predictor variable cannot be treated as an ordinal or interval measurement, we included this as a multi-categorical independent variable [43]. Indicator coding was used, which automatically generated *k*−1 dummy variables to represent the four treatments. By recoding multi-categorical variables into *k*−1 separate dummy variables, the mathematical equivalent of analysis of covariance is modelled and the linear mediation model can be estimated. The predictor variable is entered into the mediation model to quantify the indirect and direct effects of being in one treatment compared to a reference group.

To understand the indirect effect of treatment on TTE, we conducted three mediation analyses whereby one dummy variable was coded as the reference group, and the rest as comparisons. In the first, second and third analyses, SB-SOL, SB-CAP and PL-CAP were used as the reference group, respectively, which allowed us to compare the following: SB-SOL versus SB-CAP (D_1_), SB-SOL versus PL-SOL (D_2_), SB-SOL versus PL-CAP (D_3_), SB-CAP versus PL-SOL (D_4_), SB-CAP versus PL-CAP (D_5_) and PL-SOL versus PL-CAP (D_6_). Bootstrapping was set at 10,000 samples to control for Type I error [41] and bias corrected *95% CI* were calculated. An effect was significant when the CI did not contain zero. The partially standardised indirect effect (PSIE) is reported as the effect size, with values of 0.01, 0.09 and 0.25 representing small, medium, and large effect sizes, respectively [44].

## Results

### Blood Metabolites

Significant two-way interaction effects (treatment * time) were observed for bicarbonate (*F*(6, 66) = 49.688, *η*_*p*_^*2*^ = 0.819, *p* < 0.001), pH (*F*(6, 66) = 12.664, *η*_*p*_^*2*^ = 0.535, *p* < 0.001), base excess (*F*(6, 66) = 40.463, *η*_*p*_^*2*^ = 0.786, *p* < 0.001) and lactate (*F*(2.791, 30.702) = 9.975, *η*_*p*_^*2*^ = 0.476, *p* < 0.001). Bicarbonate, pH and base excess were higher pre-exercise for SB-SOL and SB-CAP compared with PL-SOL and PL-CAP (*p* < 0.05; Table 1). There were no differences in pre-exercise pH or lactate between SB-SOL and SB-CAP (*p* > 0.05). However, bicarbonate (+2.2 mmol·L^−1^, *p* = 0.007, *g* = 1.04) and base excess (+1.7 mmol·L^−1^, *p* = 0.043, *g* = 0.82) were higher pre-exercise after SB-SOL compared with SB-CAP. Bicarbonate, pH, base excess and lactate were also higher post-exercise for SB-SOL and SB-CAP compared with PL-SOL and PL-CAP (*p* < 0.05; Table 1). There were no differences in post-exercise blood metabolites between SB-SOL and SB-CAP (*p* > 0.05).

**Table 1:** Mean ± SD for blood metabolites at baseline, pre-exercise and post-exercise

	SB-SOL	SB-CAP	PL-SOL	PL-CAP
pH				
Baseline	7.445 ± 0.031	7.442 ± 0.027	7.443 ± 0.021	7.434 ± 0.026
Pre-exercise	7.521 ± 0.031 *	7.521 ± 0.040 *	7.438 ± 0.030	7.447 ± 0.032
Post-exercise	7.287 ± 0.057 *	7.293 ± 0.073 *	7.226 ± 0.064	7.237 ± 0.058
Bicarbonate (mmol·L^−1^)				
Baseline	26.8 ± 1.7	26.1 ± 1.5	26.5 ± 1.5	26.6 ± 1.5
Pre-exercise	34.4 ± 2.4 *^#^	32.2 ± 1.6 *	26.4 ± 1.1	27.1 ± 1.3
Post-exercise	15.5 ± 2.6 *	15.9 ± 3.2 *	12.6 ± 2.5	13.0 ± 1.9
Base excess (mmol·L^−1^)				
Baseline	2.8 ± 1.7	2.1 ± 1.6	2.4 ± 1.3	2.5 ± 1.1
Pre-exercise	10.6 ± 2.2 *^#^	8.9 ± 1.8 *	2.5 ± 1.2	3.2 ± 1.6
Post-exercise	-9.8 ± 3.1 *	-9.3 ± 3.9 *	−13.4 ± 3.1	−12.9 ± 2.6
Lactate (mmol·L^−1^)				
Baseline	1.34 ± 0.36	1.18 ± 0.37	1.23 ± 0.47	1.11 ± 0.33
Pre-exercise	1.50 ± 0.35	1.80 ± 0.62	1.74 ± 0.40	1.70 ± 0.53
Post-exercise	14.09 ± 3.15 *	13.58 ± 2.95 *	11.26 ± 2.17	11.73 ± 2.83

*p < 0.05 versus PL-SOL and PL-CAP, ^#^*p* < 0.05 versus SB-CAP

### TTE, Expectancy and Decline in Blood Bicarbonate

**Fig. 1:**
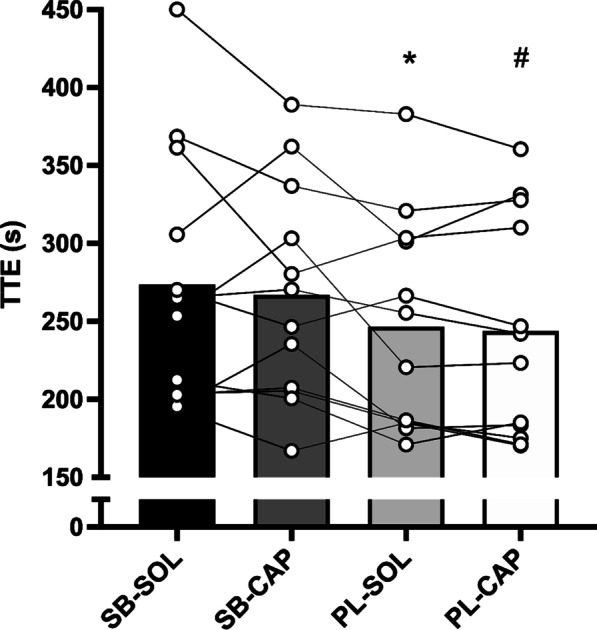
TTE cycling performance for each treatment. Data presented as mean (bar) and individual responses; * *p* < 0.05 versus SB-SOL, ^#^ *p* < 0.05 versus SB-SOL & SB-CAP

Significant main effects of treatment were observed for TTE (*F*(1.727, 18.998) = 6.147, *η*_*p*_^*2*^ = 0.358, *p* = 0.011) and decline in blood bicarbonate (*F*(3, 33) = 34.923, *η*_*p*_^*2*^ = 0.760, *p* < 0.001). TTE was greater for SB-SOL compared with PL-SOL (+27.0 s, *95% CI*: 13.0, 40.9, *p* = 0.001, *g* = 0.34) and PL-CAP (+29.7 s, *95% CI*: 12.9, 46.6, *p* = 0.003, *g* = 0.37), and for SB-CAP compared with PL-CAP (+23.0 s, *95% CI*: 5.2, 41.0, *p* = 0.016, *g* = 0.32). There were no differences for TTE between SB-SOL and SB-CAP (*p* = 0.598) or between PL-SOL and PL-CAP (*p* = 0.561). Mean and individual responses for TTE cycling performance are show in Fig. 1**.** Decline in blood bicarbonate was higher for SB-SOL compared with PL-SOL (+5.1 mmol·L^−1^, *p* < 0.001, *g* = 1.64) and PL-CAP (+4.8 mmol·L^−1^, *p* < 0.001, *g* = 1.58), and for SB-CAP compared with PL-SOL (+2.4 mmol·L^−1^, *p* = 0.001, *g* = 0.84) and PL-CAP (+2.2 mmol·L^−1^; *p* = 0.001; *g* = 0.77). Decline in blood bicarbonate was also greater for SB-SOL compared with SB-CAP (+2.7 mmol·L^−1^, *p* = 0.001, *g* = 0.79). There were no differences for decline in blood bicarbonate between PL-SOL and PL-CAP (*p* = 0.598). Mean and individual responses for decline in blood bicarbonate during TTE cycling are shown in Table 2.

**Table 2:** Mean and individual responses for decline in blood bicarbonate

Participant	Decline in blood bicarbonate during TTE cycling (mmol·L^−1^)			
	SB-SOL	SB-CAP	PL-SOL	PL-CAP
1	− 12.8*	− 8.9	− 9.7	− 8.2
2	− 21.6*	− 15.5	− 14.5	− 14.3
3	− 18.5	− 19.6*	− 16.6	− 14.4
4	− 14.9	− 15.7*	− 13.8	− 13.8
5	− 24.2*	− 20.6	− 17.5	− 17.2
6	− 17.7*	− 15.1	− 15.7	− 14.6
7	− 21.4*	− 16.9	− 13.3	− 15.4
8	− 18.3*	− 16.0	− 14.0	− 14.8
9	− 24.0*	− 20.1	− 16.0	− 17.6
10	− 19.9*	− 17.3	− 11.6	− 13.6
11	− 16.6*	− 15.5	− 11.1	− 13.7
12	− 17.5*	− 14.4	− 12.6	− 12.1
Mean	− 19.0	− 16.3	− 13.9	− 14.1
SD	3.5	3.1	2.4	2.4

*Largest decline in bicarbonate for each participant

A significant effect of treatment was observed for expectancy (*χ*^2^(3) = 15.370, *p* = 0.002). Expectancy was highest for SB-SOL (Median: 3.5) compared with SB-CAP (*Z* = 2.135, *p* = 0.033, *r* = 0.61), PL-SOL (*Z* = 3.004, *p* = 0.003, *r* = 0.87) and PL-CAP (*Z* = 2.451, *p* = 0.014, *r* = 0.71). There were no differences in expectancy for SB-CAP (Median: 2.5) compared with PL-SOL (*Z* = 0.870, *p* = 0.385) and PL-CAP (*Z* = 0.316, *p* = 0.752), or between PL-SOL and PL-CAP (Median: 2.0 vs. 2.0, *Z* = −0.553, *p* = 0.580).

### Indirect Effects of Expectancy and Decline in Blood Bicarbonate

**Fig. 2:**
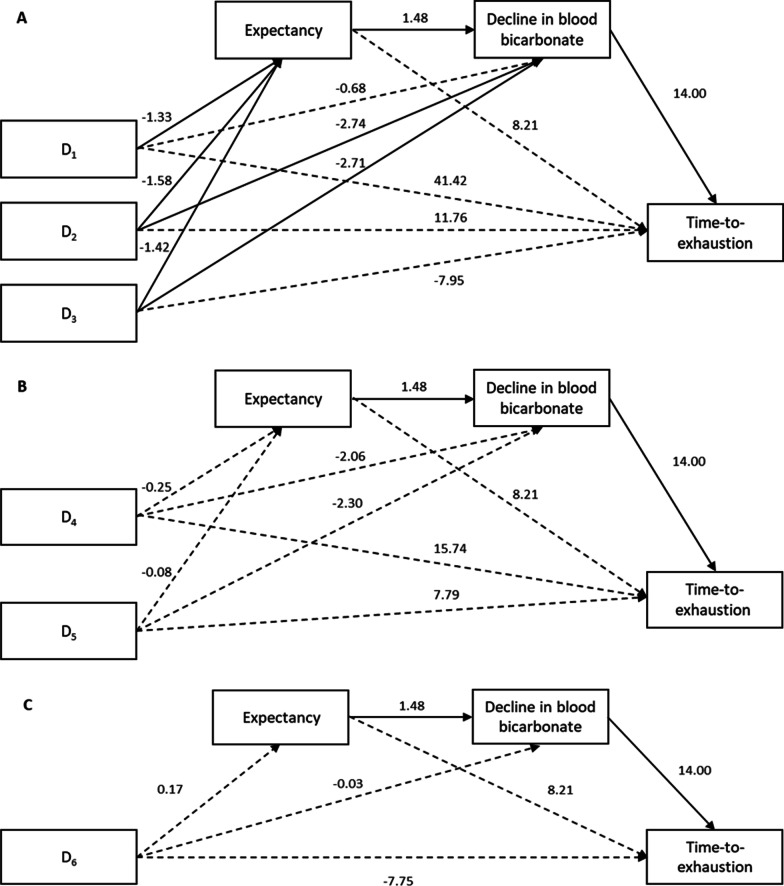
**A–C** The effects of treatment on TTE and the mediating role of expectancy and decline in blood bicarbonate. *Note:* Values presented are the unstandardised regression coefficients. A solid line represents a significant relationship. **A** Reference group = SB-SOL. D_1_ = SB-SOL versus SB-CAP, D_2_ = SB-SOL versus PL-SOL, D_3_ = SB-SOL versus PL-CAP. **B** Reference group = SB-CAP. D_4_ = SB-CAP versus PL-SOL, D_5_ = SB-CAP versus PL-CAP. **C** Reference group = PL-SOL. D_6_ = PL-SOL versus PL-CAP

Results of the mediation analyses are shown in Table 3 and Fig. 2A–C. In all analyses, no direct effects of treatment on TTE were shown (Fig. 2**A–C**). Instead, when compared to SB-CAP, PL-SOL and PL-CAP, SB-SOL had a significant indirect effect on TTE via expectations and decline in blood bicarbonate (Table 3). Similarly, when compared to PL-SOL and PL-CAP, SB-CAP was indirectly related to TTE via decline in blood bicarbonate (Table 3). No indirect effects were shown for PL-CAP or PL-SOL via expectation or decline in blood bicarbonate on TTE (Table 3). In short, participants reported higher expectations about the effectiveness of SB-SOL, which resulted in a higher decline in blood bicarbonate, and in turn, improved TTE cycling performance, whereas improvements for SB-CAP were the result of decline in blood bicarbonate. For PL-SOL and PL-CAP, expectation and decline in blood bicarbonate were not directly or indirectly related to TTE.

**Table 3:** Indirect effects of expectancy and decline in blood bicarbonate on TTE

Pathway	b (SE)	95% CI	PSIE (SE)	95% CI
*Indirect effects on TTE via expectancy*				
D_1_	− 10.95 (14.3)	− 40.50 to 16.58	− 0.15 (0.20)	− 0.57 to 0.23
D_2_	− 13.00 (17.25)	− 49.83 to 17.65	− 0.18 (0.24)	− 0.71 to 0.24
D_3_	− 11.63 (15.55)	− 45.35 to 16.16	− 0.16 (0.22)	− 0.64 to 0.22
D_4_	− 2.05 (15.49)	− 18.16 to 5.67	− 0.03 (0.08)	− 0.25 to 0.08
D_5_	− 0.68 (5.37)	− 14.48 to 8.34	− 0.01 (0.08)	− 0.20 to 0.12
D_6_	1.37 (4.96)	− 7.1 to 14.62	0.02 (0.07)	− 0.10 to 0.20
*Indirect effects on TTE via decline in blood bicarbonate*				
D_1_	− 9.45 (18.29)	− 46.51 to 26.73	− 0.13 (0.25)	− 0.64 to 0.38
**D**_**2**_	− **38.33 (20.39)**	− **81.49 to **− **2.15**	− **0.53 (0.28)**	− **1.12 to **− **0.03**
**D**_**3**_	− **37.93 (18.87)**	− **76.18 to **− **2.61**	− **0.53 (0.26)**	− **1.04 to **− **0.04**
D_4_	− 28.88 (15.49)	− 63.25 to 1.75	− 0.40 (0.21)	− 0.88 to 0.02
**D**_**5**_	− **28.48 (15.12)**	− **61.49 to **− **0.73**	− **0.39 (0.21)**	− **0.84 to **− **0.01**
D_6_	0.39 (12.29)	− 23.04 to 25.96	0.01 (0.17)	− 0.31 to 0.37
*Indirect effects on TTE via expectancy and decline in blood bicarbonate*				
**D**_**1**_	− **27.64 (15.08)**	− **63.68 to **− **5.63**	− **0.38 (0.20)**	− **0.84 to **− **0.09**
**D**_**2**_	− **32.83 (16.10)**	− **71.32 to **− **8.33**	− **0.45 (0.20)**	− **0.92 to **− **0.13**
**D**_**3**_	− **29.37 (15.05)**	− **65.97 to **− **6.75**	− **0.41 (0.19)**	− **0.86 to **− **0.10**
D_4_	− 5.18 (8.47)	− 23.18 to 10.98	−0.07 (0.11)	− 0.31 to 0.15
D_5_	− 1.73 (8.72)	− 19.48 to 16.59	− 0.02 (0.12)	− 0.26 to 0.23
D_6_	3.46 (8.12)	− 12.50 to 21.95	0.05 (0.11)	− 0.17 to 0.30

Unstandardised coefficients are shown

Bold indicates difference (*p* < 0.05)

*SE* standard error, *PSIE* partially standardized indirect effect, *TTE* time-to-exhaustion

D_1_ = SB-SOL versus SB-CAP, D_2_ = SB-SOL versus PL-SOL, D_3_ = SB-SOL versus PL-CAP, D_4_ = SB-CAP versus PL-SOL, D_5_ = SB-CAP versus PL-CAP, D_6_ = PL-SOL versus PL-CAP

### Gastrointestinal Discomfort

All participants reported GI discomfort following SB-SOL and SB-CAP, with ‘gut fullness’ the most common side-effect. There were no differences in aggregate GI discomfort between treatments at 30 min (*χ*^2^(3) = 3.051, *p* = 0.351), 60 min (*χ*^2^(3) = 5.250, *p* = 0.154), pre-exercise (*χ*^2^(3)=5.797, *p* = 0.122) and post-exercise (*χ*^2^(3) = 2.971, *p* = 0.396).

## Discussion

The aims of our retrospective study were (1) to determine the influence of expectancy and decline in blood bicarbonate following NaHCO_3_ administered in solution and capsules on TTE cycling performance, and (2) examine if changes in performance are mediated by the expectation of receiving NaHCO_3_ and decline in blood bicarbonate. Administering NaHCO_3_ in solution and capsules were indirectly related to TTE cycling performance via decline in blood bicarbonate, while giving NaHCO_3_ in solution resulted in greater expectations that it would improve performance. Our mediation analyses also revealed an indirect relationship between expectancy and decline in blood bicarbonate on TTE cycling performance when NaHCO_3_ was administered in solution, but not for the other treatments. These findings suggest that participants’ reporting greater expectancy after consuming NaHCO_3_ in solution may subconsciously exert themselves harder during an exercise task due to psychologically manipulated expectations (i.e., reduced feelings of fatigue). In turn, this is likely to lead to a greater decline in blood bicarbonate that may indicate an enhanced extracellular buffering response that contributes to improvement in performance.

In agreement with the proposed ergogenic effect of acute NaHCO_3_ ingestion on TTE cycling performance [10–12], SB-SOL and SB-CAP significantly improved TTE cycling performance compared to placebo treatments in our cohort of male and female recreationally trained cyclists (Fig. 1). Recent evidence suggests that the effect of NaHCO_3_ on high-intensity exercise performance could be gender-specific [45], however given our small sample size of female participants (n = 3) it was not possible to further explore this argument. Whilst this is the first study to compare the benefits of NaHCO_3_ given in solution and capsules on TTE cycling performance, it seems that researchers using solution ingestion approaches are more likely to report improvements than those opting for capsules [13–15]. We recently demonstrated that participants perceive solution NaHCO_3_ beverages to have a ‘worse’ taste than NaHCO_3_ administered in capsules, which sometimes results in them successfully identifying what treatment has been given [28]. Other researchers have suggested that taste alone can be ergogenic, owing to centrally acting mechanisms that alter participants’ perception of effort [46, 47]. Since perception of effort is a primary determinant of exercise performance in motivated individuals [48, 49], it is likely that participants’ expectations that they have received NaHCO_3_ influences ergogenic benefits. We argue that participants able to detect NaHCO_3_ experience higher response expectancies (i.e., prediction that exercise will be easier) that in turn, increase their belief that they can exert more effort during an exercise task [26]. Our results highlight that the administration of NaHCO_3_ in solution may therefore overestimate its ‘true’ efficacy during randomised controlled trials whereby psychological components could also contribute to benefits [13–15]. It is important that researchers adequately blind participants from the treatment given to provide a more accurate inference to the pharmacological effects of NaHCO_3_ on sports performance outcomes.

As expected, participants’ expectancy was highest for SB-SOL compared to the other treatments, likely due to the ‘poor’ taste of solution NaHCO_3_ beverages and GI side-effects weakening blinding efficacy [28], which subsequently increased participants’ expectation of positive outcomes. Participants’ expectancy is believed to induce placebo effects [16], but as we did not directly deceive participants about which treatment had been received, instead evaluating whether differences in acute NaHCO_3_ ingestion strategy altered expectancy, it is possible that some participants were unable to identify NaHCO_3_ in either solution or capsules (see Gurton et al. [28] for data). This may have limited participants’ expectation about the effect of NaHCO_3_ and thus, reduced any potential influence on TTE cycling performance. Since response expectancies are one of the psychological components believed to contribute towards placebo effects [25], it is crucial researchers measure participants’ expectation when evaluating the efficacy of acute NaHCO_3_ ingestion for improving sports performance.

Administering NaHCO_3_ in solution and capsules elevated acid-base balance pre- and post-exercise compared to placebo treatments (Table 1). Achieving an >5 mmol·L^−1^ increase in blood bicarbonate is considered crucial for maximising performance benefits after acute ingestion of 0.3 g·kg^−1^ BM NaHCO_3_ [1], but improvements have still been shown for alternative supplementation approaches (i.e., chronic and smaller dosing strategies) despite changes in blood bicarbonate failing to reach this ‘ergogenic’ threshold [50, 51]. In these circumstances, it is possible that decline in blood bicarbonate during exercise is more important, as this directly infers whether enhanced buffering response for NaHCO_3_ was fully used throughout an exercise task [24]. Decline in blood bicarbonate during TTE cycling was greater for SB-SOL and SB-CAP than their respective placebo treatments, with highest decline reported following SB-SOL in 83% of participants and SB-CAP in 17% of participants (Table 2). Importantly, we also found that decline in blood bicarbonate was greater for NaHCO_3_ administered in solution than capsules. According to arguments made by da Silva et al. [52], if total blood volume is ~5 L, then as decline in blood bicarbonate was ~3.0 mmol·L^−1^ higher for SB-SOL compared with SB-CAP, we can assume that that an extra ~15 mmoles of H^+^ could have been neutralised (based on the 1:1 stoichiometry of bicarbonate buffering system). Whilst post-exercise blood lactate was elevated for both NaHCO_3_ treatments compared with placebos, there were no differences between SB-SOL and SB-CAP. Despite enhanced extracellular buffering potential for SB-SOL, these results seem to suggest that lactate-H^+^ cotransport via the monocarboxylate transporter 1/4 was not further augmented by giving NaHCO_3_ in solution [8]. Our findings reinforce arguments by Higgins et al. [11] that enhanced TTE cycling performance after NaHCO_3_ is not solely contributed to augmented metabolic flux, however further research is needed to understand the influence of decline in blood bicarbonate on the performance enhancing effects of acute and chronic NaHCO_3_ supplementation.

This was the first study to examine the mediating role of expectancy and decline in blood bicarbonate on the ergogenic benefits of NaHCO_3_ (Fig. 2A–C). Firstly, whilst expectancy was increased for SB-SOL, it was not directly related to TTE performance (Table 3). Previous studies that demonstrated positive expectancy effects of NaHCO_3_ on exercise performance employed standardized scripts that deceived participants about which treatment had been given and outlined the ‘proven’ ergogenicity of NaHCO_3_ [19, 20]. These methodological differences likely increased expectancy compared to our study [26], as some participants would have been unaware of which treatment had been given, or the potential benefits for NaHCO_3_. Secondly, both SB-SOL and SB-CAP were indirectly related to TTE cycling performance via decline in blood bicarbonate, suggesting this could predict the ergogenic effects of NaHCO_3_. It is logical to theorize that participants who report greatest decline in blood bicarbonate would be more likely to see improvements in performance, as this indicates whether enhanced extracellular buffering for NaHCO_3_ was utilised [24]. Notably, there was also an indirect relationship between expectancy and decline in blood bicarbonate on TTE performance for SB-SOL, but not SB-CAP (Table 3). In other words, participants with the highest expectation (i.e., prediction that exercise will be ‘easier’) for SB-SOL may have exerted themselves harder during TTE cycling, which augmented physiological response resulting in a greater decline in blood bicarbonate. As such, we argue that these participants were able to capitalise on both the psychological and physiological effects of NaHCO_3_ and in turn, improve TTE cycling performance to a larger extent than when one is absent [27]. Improvements during TTE cycling for SB-SOL were therefore influenced by participants’ expectations [16, 25], whereas improvements for SB-CAP can be attributed to NaHCO_3_ pharmacological properties augmenting blood bicarbonate buffering capacity. These findings from our mediation analyses reinforce that administering NaHCO_3_ in solution may overestimate its ‘true’ efficacy during randomised controlled trials because of participants’ expectancy that the treatment would improve their performance [17, 18]. Our study bridges the gap between placebo effect and sports performance research by offering an alternative explanation for the equivocal effect of NaHCO_3_ on TTE cycling performance. Considering these findings, we recommend that researchers administer NaHCO_3_ in capsules when examining sports performance outcomes, allowing them to attribute ergogenic benefits to treatment efficacy instead of placebo effects such as response expectancy.

There are methodological limitations that need to be considered when interpreting results and should be addressed in the future. We did not include a control group (i.e., no treatment given) during the present study, which would have helped better understand the effects of expectations on TTE cycling performance [53]. We also chose to administer treatments 90 min pre-exercise, as this time-frame is believed to elicit an almost certain increase in blood bicarbonate >5 mmol·L^−1^ (~97% probability) [54]. Despite this, we found that pre-exercise blood bicarbonate was ~2.0 mmol·L^−1^ higher for SB-SOL versus SB-CAP. Considering that peak changes in blood bicarbonate following 0.3 g·kg^−1^ BM NaHCO_3_ typically occur later when administered in capsules [54], it is possible differences for decline in blood bicarbonate between NaHCO_3_ treatments were due to participants achieving a greater alkalotic state for SB-SOL pre-exercise, as well as greater effort owing to greater expectations from identifying treatments. Future work examining decline in blood bicarbonate between different NaHCO_3_ ingestion strategies needs to adopt a time-to-peak approach to ensure pre-exercise blood bicarbonate is similar between treatments [36, 50].

## Conclusions

In this retrospective study, we found that 0.3 g·kg^−1^ BM NaHCO_3_ given in both solution and capsules improved TTE cycling performance. There was an indirect relationship between expectancy and decline in blood bicarbonate on TTE cycling for NaHCO_3_ administered in solution, but not capsules. These findings suggest that improvements in TTE cycling performance for NaHCO_3_ administered in solution were likely the result of greater response expectancies (i.e., predictions that exercise would be easier) and extracellular buffering response, whereas benefits for NaHCO_3_ given in capsules were due to pharmacological properties augmenting blood bicarbonate buffering capacity. Participants able to detect that NaHCO_3_ has been given may exert themselves harder during exercise, which in turn allows them to capitalise on both the psychological and physiological effects of NaHCO_3_, subsequently leading to increased performance benefits compared with when only one is present. In light of our findings, we recommend researchers examining potential ergogenic benefits for NaHCO_3_ adopt capsule ingestion strategies to ensure positive effects can be attributed to treatment efficacy instead of participants’ expectations.

## Data Availability

The datasets generated during and/or analysed during the current study are available from the corresponding author on reasonable request.

## Footnotes

1 Participants included in a previous study by Gurton et al. [28] titled “efficacy of sodium bicarbonate ingestion strategies for protecting blinding”. This study had separate aims, which were to compare the blinding efficacy of various sodium bicarbonate ingestion strategies. Published within European Journal of Applied Physiology (September 2022).
